# Development and validation of a nomogram for predicting 5-year progression-free survival probability in differentiated thyroid cancer patients with lung metastases

**DOI:** 10.3389/fendo.2026.1938835

**Published:** 2026-10-21

**Authors:** Congcong Wang, Xiaonian Zhang, Yingying Zhang, Jingshuang Liu, Guohua Qin, Peihang Han, Yutian Li, Renfei Wang, Xufu Wang

**Affiliations:** 1 Department of Nuclear Medicine, The Affiliated Hospital of Qingdao University, Qingdao, Shandong, China; 2 Department of Emergency Medicine, The Affiliated Hospital of Qingdao University, Qingdao, Shandong, China; 3 Department of Radiology, Qingdao Women and Children’s Hospital, Qingdao, Shandong, China; 4 Department of Nuclear Medicine, Shanghai Tenth People’s Hospital, Tongji University School of Medicine, Shanghai, China

**Keywords:** differentiated thyroid cancer, lung metastases, nomogram model, progression-free survival, progressive disease, radioiodine therapy

## Abstract

**Background and objective:**

Progressive disease (PD) in differentiated thyroid cancer patients with lung metastases (DTC-LM) poses a significant clinical challenge, yet reliable predictive tools remain limited. This study aimed to develop and validate a nomogram for individualized prediction of 5-year progression-free survival (PFS) probability in DTC-LM patients.

**Methods:**

This retrospective study included 284 DTC-LM patients from two institutions, divided into a training cohort (Qingdao cohort, n = 184) and an external validation cohort (Shanghai cohort, n = 100). Patients were classified as having PD or non-PD (NPD) at final follow-up. Univariable and multivariable Cox proportional hazards regression analyses were used to identify independent predictors of PD. A nomogram based on the multivariable Cox model was developed to estimate 5-year PFS probability. Model performance was evaluated using the C-index, calibration curves, and decision curve analysis (DCA).

**Results:**

Multivariate analysis identified age upon diagnosis (≥55 years), tumor size, pre-ablation stimulated thyroglobulin (Ps-Tg ≥528.5 ng/mL), and initial RAI avidity status as independent predictors of PD. The 5-year PFS probability nomogram demonstrated excellent discrimination, with a C-index of 0.874 in the training cohort and 0.769 in the external validation cohort. Calibration curves showed good agreement between predicted and observed outcomes, and DCA confirmed favorable clinical net benefit.

**Conclusion:**

This nomogram incorporating four readily available clinical variables showed good discrimination for predicting 5-year PFS probability in DTC-LM patients and may facilitate preliminary risk stratification and timely therapeutic decision-making.

## 1 Introduction

Thyroid cancer is one of the most common malignancies of the endocrine system, with its incidence steadily rising worldwide (1, 2). Differentiated thyroid cancer (DTC), which encompasses papillary thyroid cancer (PTC) and follicular thyroid cancer (FTC), accounts for over 90% of all thyroid cancers (3). Following total thyroidectomy, selective radioiodine (RAI) therapy, and thyroid-stimulating hormone (TSH) suppressive therapy, most DTC patients achieve a favorable prognosis, with 10-year survival rates exceeding 90% (4). However, approximately 4-23% of DTC patients develop distant metastases (DM) during the course of their disease, with the lungs being the most frequently involved site (5).

Compared with non-metastatic patients, those with lung metastases (DTC-LM) have a considerably poorer prognosis. For DTC-LM patients, 10-year survival rates range from approximately 60% to 88%, depending on various clinical and treatment-related factors (6, 7). Alarmingly, among DTC-LM patients, approximately two-thirds may eventually progress to radioiodine-refractory DTC (RAIR-DTC). Once DTC-LM progresses to RAIR-DTC, the prognosis worsens dramatically, with a 10-year survival rate of less than 10% (8, 9). RAIR-DTC represents the most common cause of thyroid cancer-related deaths and poses a major challenge in clinical management.

Given the limited benefit and increased risk of adverse effects from repeated RAI therapy in patients who are refractory or who have developed progressive disease (PD), early identification of patients at risk of PD is critical, both to avoid unnecessary RAI therapy and to enable timely initiation of alternative treatments, such as multi-kinase inhibitors (MKIs). However, predicting which DTC-LM patients will develop PD remains challenging. Currently, the diagnosis of PD in DTC-LM patients is primarily based on long-term follow-up imaging and biochemical assessments, which are time-consuming and often lead to delayed therapeutic decisions.

In recent years, various clinicopathological and molecular factors have been identified as potential predictors of PD in DTC-LM, including older age at diagnosis, extrathyroidal extension (ETE), larger primary tumor size, and BRAF^V600E^ mutation (10–12). However, individual factors alone have shown limited predictive performance. The nomogram, as an effective predictive tool that integrates the weighted contributions of multiple independent risk factors, has been increasingly applied in oncological research to provide individualized risk probability estimates for specific clinical events. While several nomogram models have been developed to predict distant metastasis or survival outcomes in DTC patients, few studies have specifically focused on predicting PD in DTC-LM patients, and even fewer have integrated serological markers such as pre-ablation stimulated thyroglobulin (Ps-Tg) into the predictive model.

In this study, we aimed to develop and validate a nomogram model for predicting the probability of 5-year progression-free survival (PFS) in DTC-LM patients, incorporating comprehensive clinicopathological variables, genetic markers, imaging characteristics, and serological biomarkers including Ps-Tg. By providing an individualized, clinically applicable risk prediction tool, we hope to facilitate early identification of high-risk patients and enable more timely and personalized therapeutic decision-making.

## 2 Materials and methods

### 2.1 Patients

This retrospective study analyzed medical records and follow-up data from DTC-LM patients who underwent total or subtotal thyroidectomy followed by RAI therapy at the Department of Nuclear Medicine, the Affiliated Hospital of Qingdao University between January 2006 and December 2024, and at the Department of Nuclear Medicine, Shanghai Tenth People’s Hospital between January 2016 and December 2020. The inclusion criteria were: (1) total or subtotal thyroidectomy with selective cervical lymph node dissection; (2) histopathologically confirmed DTC; (3) at least one course of standardized RAI therapy; (4) diagnosis of LM; and (5) age≥18 years at the time of diagnosis. Patients were excluded if they had positive anti-thyroglobulin antibodies (TgAb), concurrent malignancies, missing critical clinical or follow-up data, or had received other anti-tumor therapies. The study protocol was approved by the Ethics Committee of the Affiliated Hospital of Qingdao University and was conducted in accordance with the Declaration of Helsinki. Written informed consent was exempted by the institutional review board (Ethics approval number: QYFY WZLL 50357). The training cohort was drawn from the Affiliated Hospital of Qingdao University (Qingdao cohort), whereas the external validation cohort was drawn from Shanghai Tenth People’s Hospital (Shanghai cohort) (**Figure 1**).

### 2.2 Data collection and RAI therapy protocol

The following clinical and pathological variables were collected: age upon diagnosis, histological subtype, gender, multicentricity, primary tumor size, BRAF^V600E^ mutation status, ETE, T stage, N stage, lymph node metastasis (LNM) ratio, Ps-Tg, initial RAI avidity status, presence of multiple lung metastases (LM), and the largest diameter of LM. Initial RAI avidity status was determined based on the post-therapeutic ^131^I whole-body scan/SPECT-CT (Rx-WBS) performed 3–7 days after the initial RAI therapy. Subsequently, patients were staged according to the AJCC TNM classification (8th edition) for both T and N categories (13, 14). The diagnosis of LM was established on the basis of chest computed tomography (CT) findings, Rx-WBS results, and thyroglobulin (Tg) and TgAb levels.

All patients received one or more courses of RAI therapy after total thyroidectomy, in accordance with the American Thyroid Association (ATA) guidelines in effect at the time of treatment (i.e., the 2009 and 2015 editions) (15, 16). The administered RAI activity ranged from 150 to 200 mCi per session, with dosage adjustments made according to individual patient status. Rx-WBS and SPECT/CT were performed 3–7 days after each RAI administration. Following RAI therapy, all patients were placed on levothyroxine suppressive therapy to maintain TSH suppression. Repeat RAI therapies were administered at 6- to 12-month intervals, provided that the disease continued to demonstrate RAI avidity and showed a favorable clinical response (17).

### 2.3 Laboratory assays for Tg and TgAb

Serum Tg and TgAb levels were measured using an electrochemiluminescence immunoassay according to institutional protocols (18). For Ps-Tg measurement, blood samples were collected before the initial RAI therapy after TSH stimulation (by thyroid hormone withdrawal administration). The Tg assay had a functional sensitivity of 0.04 ng/mL and a measuring range of 0.04–500 ng/mL. For samples with Ps-Tg levels exceeding the upper limit of the measuring range (>500 ng/mL), appropriate dilution (e.g., 1:10 or 1:100) was performed using the manufacturer-provided diluent, and the samples were re-measured; the final concentrations were calculated by multiplying the measured values by the corresponding dilution factors. TgAb levels exceeding 40 IU/mL were defined as positive.

### 2.4 Clinical outcome assessment

Structural tumor response was evaluated every 6–12 months after RAI therapy using the Response Evaluation Criteria in Solid Tumors version 1.1 (RECIST 1.1) (19, 20). All available imaging studies, including chest CT, Rx-WBS, and SPECT/CT when available, were independently reviewed by two board-certified nuclear medicine physicians who were blinded to each other’s assessments. Discrepancies were resolved by consensus or by a third senior nuclear medicine physician. Serum Tg and TgAb levels were used as supportive biochemical information but did not independently define structural PD.

For patients with at least one measurable lesion (≥10 mm in diameter), the structural response were defined as follows: complete response (CR), disappearance of all target lesions; partial response (PR), ≥30% decrease from baseline in the sum of the longest diameters (SLD) with no new lesions; stable disease (SD), insufficient change to qualify for PR or PD; and PD, PD was defined as a ≥20% increase in the SLD relative to the baseline with an absolute increase of at least 5 mm, or the appearance of new lesions. For patients with all LM lesions <10 mm in diameter (non-target lesions), responses were classified as CR (disappearance of all lesions and normalization of serum Tg, consistent with RECIST 1.1 provisions for non-target lesions when tumor markers are initially elevated), non-CR/non-PD (persistence of one or more lesions), or PD (unequivocal progression of existing lesions or new lesions). Serum Tg was not used to define PD. The date of the first documented PD event was recorded as the event date. Patients without documented PD were censored at death or last follow-up. For subsequent analysis, patients achieving CR, PR, or SD (among those with target lesions) and those with CR or non‐CR/non‐PD (among those with non‐target lesions) were collectively grouped as non‐progressive disease (NPD) (17).

### 2.5 Statistical analysis

Continuous variables were summarized as medians with ranges, while categorical variables were presented as frequencies and percentages. The Mann-Whitney U test was applied to compare Ps-Tg levels between the PD and NPD groups. Receiver operating characteristic (ROC) curve analysis was performed to determine the optimal Ps-Tg cutoff for predicting PD. Follow-up time was defined as the interval from the first RAI therapy to the date of first documented PD, death, or last follow-up, whichever occurred first. The primary outcome was time to first PD. Univariable and multivariable Cox proportional hazards regression models were conducted to identify prognostic factors. Variables with P < 0.1 in univariate analysis were entered into the multivariable Cox regression. Hazard ratios (HRs) and profile likelihood 95% confidence intervals (CIs) are reported. Internal optimism was quantified using 1000 bootstrap resamples to obtain the optimism-corrected C-index and calibration slope. Repeated 5-fold cross-validation (100 repetitions) was used to estimate the mean cross-validated C-index and its standard deviation.

A 5-year progression-free survival (PFS) probability nomogram was developed and validated by assessing discrimination, calibration, and clinical utility. The predictive performance of the nomogram was subsequently validated in the external validation cohort. Harrell’s concordance index (C-index) was calculated to evaluate discrimination, and time-dependent ROC curves were plotted to further assess the model’s discriminatory ability. Calibration curves were generated by comparing predicted probabilities with observed probabilities at different time points. Decision curve analysis (DCA) was performed to evaluate clinical utility and net benefit across different threshold probabilities. All statistical analyses were performed using R software (version 4.2.1; http://www.r-project.org/). A two-sided P value <0.05 was considered statistically significant.

## 3 Results

### 3.1 Characteristics of the study population

During the study period, 284 patients with DTC-LM who met the inclusion criteria were enrolled. The Qingdao cohort (n = 184) and Shanghai cohort (n = 100) were used as the training and the external validation cohorts, respectively (**Figure 1**). Baseline clinical characteristics were comparable between the two cohorts and are summarized in **Table 1**.

**Table 1:** Clinicopathologic characteristics of DTC-LM patients in training and validation cohorts.

Characteristics	n	Cohort, no. (%)		*χ^2^/U*	P value
		Training (n = 184)	Validation (n = 100)		
Gender				3.644^a^	0.056
Female	158	110 (59.8%)	48 (48.0%)		
Male	126	74 (40.2%)	52 (52.0%)		
Age upon diagnosis (years)				0.760^a^	0.383
<55	180	120 (65.2%)	60 (60.0%)		
≥55	104	64 (34.8%)	40 (40.0%)		
Histological type				1.713^a^	0.191
PTC	256	169 (91.8%)	87 (87.0%)		
FTC	28	15 (8.2%)	13 (13.0%)		
Tumor size (cm)		2.0 (1.4, 3.0)	2.0 (1.3, 3.0)	9099.5^b^	0.879
BRAF^V600E^ mutations				4.715^a^	0.095
Yes	78	43 (23.4%)	35 (35.0%)		
NO	173	117 (63.6%)	56 (56.0%)		
Unknown	33	24 (13.0%)	9 (9.0%)		
Multicentricity				3.391^a^	0.066
No	138	82 (44.6%)	56 (56.0%)		
Yes	146	102 (55.4%)	44 (44.0%)		
Ratio of LNM				3.100^a^	0.078
<50%	173	119 (64.7%)	54 (54.0%)		
≥50%	111	65 (35.3%)	46 (46.0%)		
ETE				0.763^a^	0.382
No	112	76 (41.3%)	36 (36.0%)		
Yes	172	108 (58.7%)	64 (64.0%)		
T stage				0.965^a^	0.326
T1-T3a	119	81 (44.0%)	38 (38.0%)		
T3b-T4	165	103 (56.0%)	62 (62.0%)		
N stage				0.937^a^	0.333
N1b	239	152 (82.6%)	87 (87.0%)		
N0-N1a	45	32 (17.4%)	13 (13.0%)		
Ps-Tg (ng/mL)		130.9 (38.6, 592.6)	138.5 (53.6, 668.0)	8763.0^b^	0.509
Initial RAI avidity status				2.657^a^	0.103
RAI avidity	175	107 (58.2%)	68 (68.0%)		
Partial or no RAI avidity	109	77 (41.8%)	32 (32.0%)		
Largest size of LM				1.340^a^	0.247
<10 mm	198	124 (67.4%)	74 (74.0%)		
≥10 mm	86	60 (32.6%)	26 (26.0%)		
Multiple LM lesions				0.787^a^	0.375
No	86	59 (32.1%)	27 (27.0%)		
Yes	198	125 (67.9%)	73 (73.0%)		
Follow-up period (months)		75.9 (40.5-109.1)	80.5 (48.0-123.4)	8319.0^b^	0.183

DTC, differentiated thyroid cancer; LM, lung metastases; PTC, papillary thyroid cancer; FTC, follicular thyroid cancer; LNM, lymph node metastasis; ETE, extrathyroidal extension; T, tumor; N, node; Ps-Tg, pre-ablation stimulated thyroglobulin; RAI, radioiodine; NA, Not applicable. ^a^means chi-squared test; ^b^means Mann-Whitney *U* test.

**Figure 1:**
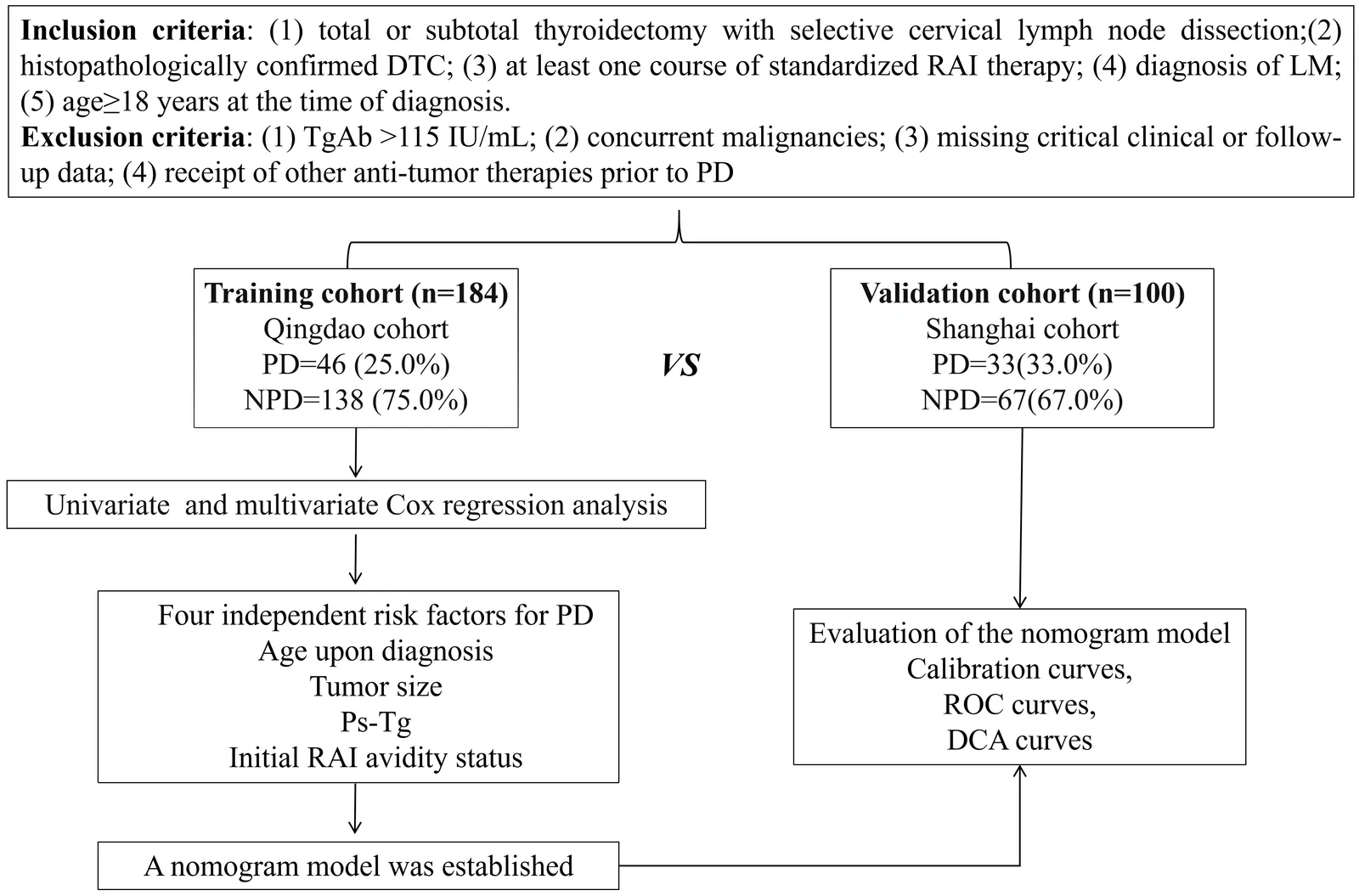
Flowchart of patient selection and study design for DTC-LM patients. DTC, differentiated thyroid cancer; LM, lung metastasis; TgAb, thyroglobulin antibody; PD, progressive disease; NPD, non-progressive disease; Ps-Tg, pre-ablation stimulated thyroglobulin; RAI, radioiodine; ROC, receiver operating characteristic; DCA, decision curve analysis.

### 3.2 Clinical outcomes of DTC-LM patients in the training and validation cohorts

The median follow-up duration was 75.9 months (IQR, 40.5–109.1) in the training cohort and 80.5 months (IQR, 48.0–123.4) in the external validation cohort. At the last follow‐up, the proportions of patients with PD and NPD in the training cohort were 25.0% (n = 46) and 75.0% (n = 138), respectively. In the external validation cohort, the corresponding rates were 33.0% (n = 33) and 67.0% (n = 67). The PD rate did not differ significantly between the two cohorts (**Figure 2**).

**Figure 2:**
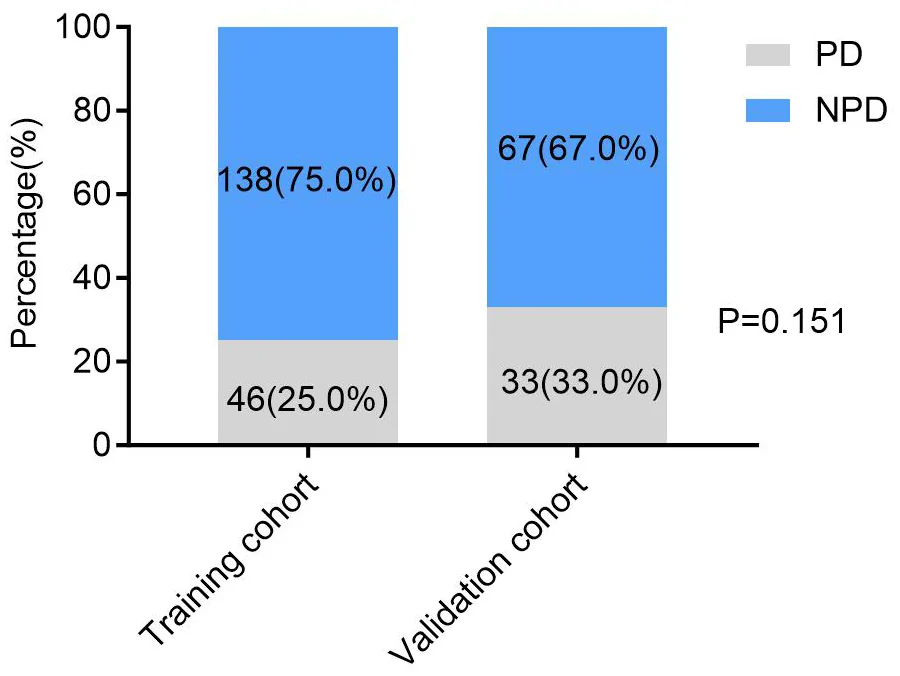
Clinical outcomes (PD vs. NPD) of DTC-LM patients in the training and validation cohorts. PD, progressive disease; NPD, non-progressive disease.

### 3.3 Predictive performance of Ps-Tg for PD in the training cohort of DTC-LM patients

The median Ps-Tg level in the PD group was 1017.5 ng/mL (IQR, 347.7-2486.5), significantly higher than the 87.0 ng/mL (IQR, 33.8-262.2) observed in the NPD group (U = 1125.0, P < 0.001). ROC curve analysis (**Figure 3**) identified an optimal Ps-Tg cutoff of 528.5 ng/mL for PD prediction, yielding a sensitivity of 69.6%, a specificity of 87.7%, and an AUC of 0.823.

**Figure 3:**
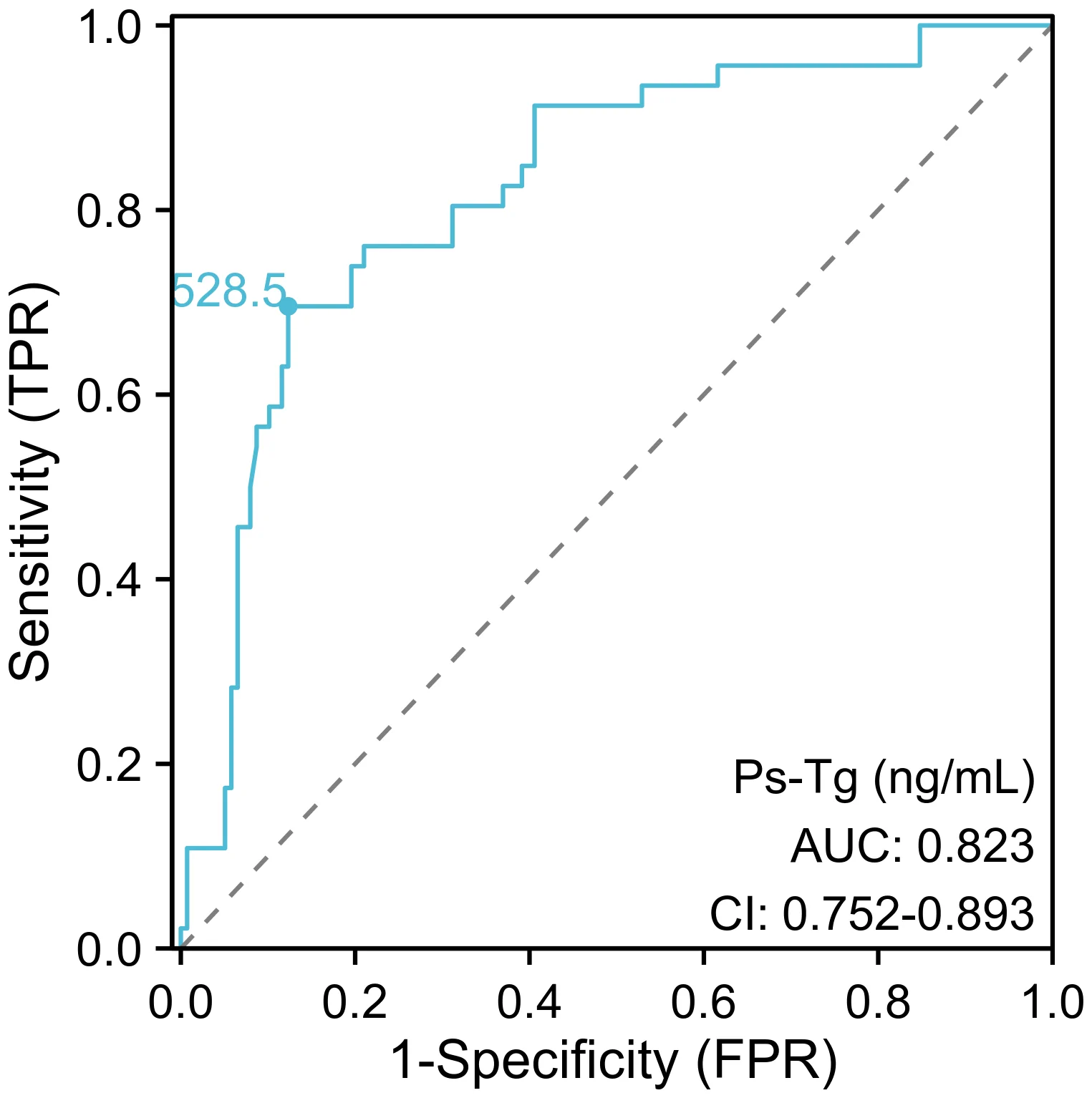
ROC curves of Ps-Tg for detecting PD in DTC-LM patients in the training cohort. Ps-Tg: pre-ablation stimulated thyroglobulin; PD: progressive disease; NPD, non-progressive disease; ROC, receiver operating characteristic; AUC, area under the ROC curve; *** P<0.001.

### 3.4 Risk factors for PD in the training cohort

To explore the relationship between clinical characteristics and PD in DTC‐LM patients, 14 variables were entered into Cox analysis (**Table 2**). This initial screening identified the following factors as significantly associated with PD: gender (P = 0.033), age upon diagnosis (P < 0.001), tumor size (P < 0.001), ETE (P = 0.001), T stage (P < 0.001), Ps-Tg (P < 0.001), initial RAI avidity status (P < 0.001), largest size of LM (P < 0.001), and multiple LM lesions (P = 0.023). Variables with P < 0.10 were entered into the multivariable Cox regression model. After backward stepwise selection based on AIC, age upon diagnosis (<55 years/≥55 years) (Hazard ratio [HR]: 3.374, 95% confidence interval [CI]: 1.670 - 6.816, P < 0.001), tumor size (HR: 1.264, 95% CI: 1.003 - 1.593, P = 0.047), Ps-Tg (<528.5 ng/mL/≥528.5 ng/mL) (HR: 2.709, 95% CI: 1.210 - 6.065, P = 0.015), and initial RAI avidity status (RAI avidity/Partial or no RAI avidity) (HR: 8.462, 95% CI: 2.407 - 29.753, P < 0.001) as independently associated with PD risk (**Table 2**).

**Table 2:** Univariate and multivariate Cox regression analysis for predictors of PD in the training cohort of DTC-LM patients.

Characteristics	Total(N)	Univariate analysis		Multivariate analysis	
		Hazard ratio (95% CI)	P value	Hazard ratio (95% CI)	P value
Gender	184				
Male	74	Reference		Reference	
Female	110	0.527 (0.293 - 0.948)	**0.033**	0.618 (0.295 - 1.296)	0.203
Age upon diagnosis (years)	184				
<55	120	Reference		Reference	
≥55	64	3.574 (1.963 - 6.507)	**< 0.001**	3.374 (1.670 - 6.816)	**< 0.001**
Histological type	184				
PTC	169	Reference			
FTC	15	2.008 (0.894 - 4.514)	0.091		
BRAF^V600E^ mutations	184				
Unknown	24	Reference		Reference	
No	117	0.653 (0.261 - 1.633)	0.362	0.567 (0.203 - 1.578)	0.277
Yes	43	2.271 (0.900 - 5.732)	0.082	1.432 (0.477 - 4.301)	0.522
Multicentricity	184				
Yes	102	Reference			
No	82	1.196 (0.666 - 2.146)	0.550		
Tumor size (cm)	184	1.393 (1.206 - 1.608)	**< 0.001**	1.264 (1.003 - 1.593)	**0.047**
Ratio of LNM	184				
<50%	119	Reference			
≥50%	65	0.776 (0.416 - 1.449)	0.426		
ETE	184				
Yes	108	Reference		Reference	
No	76	0.305 (0.147 - 0.634)	**0.001**	5.566 (3.020 - 7.893)	0.697
T stage	184				
T3b-T4	103	Reference		Reference	
T1-T3a	81	0.275 (0.133 - 0.572)	**< 0.001**	1.023 (0.158 - 4.124)	0.196
N stage	184				
N1b	152	Reference			
N0-N1a	32	0.778 (0.347 - 1.742)	0.542		
Ps-Tg (ng/mL)	184				
<528.5	135	Reference		Reference	
≥528.5	49	8.748 (4.649 - 16.458)	**< 0.001**	2.709 (1.210 - 6.065)	**0.015**
Initial RAI avidity status	184				
RAI avidity	107	Reference		Reference	
Partial or no RAI avidity	77	24.359 (7.553 - 78.558)	**< 0.001**	8.462 (2.407 - 29.753)	**< 0.001**
Largest size of LM	184				
<10 mm	124	Reference		Reference	
≥10 mm	60	2.988 (1.667 - 5.357)	**< 0.001**	1.026 (0.507 - 2.078)	0.943
Multiple LM lesions	184				
Yes	125	Reference		Reference	
No	59	0.428 (0.206 - 0.891)	**0.023**	0.579 (0.251 - 1.339)	0.201

PD, progressive disease; DTC, differentiated thyroid cancer; LM, lung metastases; PTC, papillary thyroid cancer; FTC, follicular thyroid cancer; LNM, lymph node metastasis; ETE, extrathyroidal extension; T, tumor; N, node; Ps-Tg, pre-ablation stimulated thyroglobulin; RAI, radioiodine; NA, Not applicable. Bold values indicate statistically significant P values (P < 0.05).

### 3.5 Prognostic nomogram for 5-year PFS probability in DTC-LM patients

A nomogram was constructed to estimate the 5-year PFS probability in DTC‐LM patients, incorporating the independent risk factors identified by multivariate Cox regression. For each patient, points were assigned to the constituent variables and summed to generate a total score, which was then projected vertically onto the risk axis to derive the predicted 5-year PFS probability. Regarding the relative contribution of each predictor, initial RAI avidity status exerted the greatest influence on 5-year PFS probability, followed by tumor size, Ps-Tg, and age upon diagnosis (**Figure 4**). The nomogram is intended for use immediately after the first Rx-WBS following initial RAI, when age upon diagnosis, tumor size, Ps-Tg, and initial RAI avidity status are all available.

**Figure 4:**
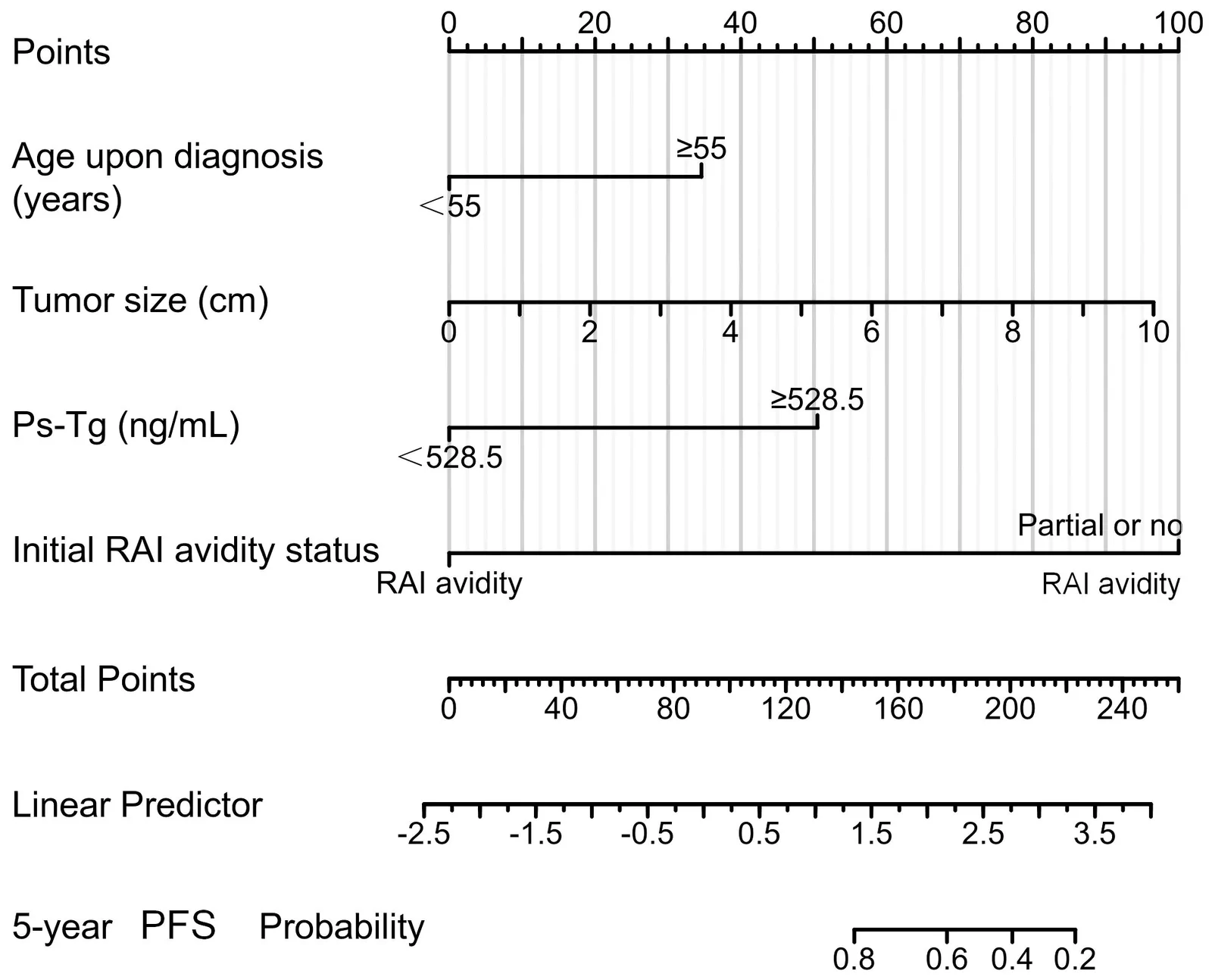
Nomogram for predicting the 5-year PFS probability in patients with DTC-LM. Interpretation Guide: To apply this nomogram to an individual patient, first locate the patient’s values for each predictor (age upon diagnosis, tumor size, Ps-Tg, and initial RAI avidity status) on their respective variable axes. For each variable, draw a vertical line upward to the top “Points” axis and record the corresponding score. Sum all scores to obtain the total points, then find this total on the bottom “Total Points” axis. Finally, draw a vertical line downward from that point to the “Risk” axis to read the predicted the 5-year PFS probability. PFS, progression-free survival (PFS); Ps-Tg, pre-ablation stimulated thyroglobulin; DTC, differentiated thyroid cancer; LM, lung metastases; RAI, radioiodine.

### 3.6 External validation of the predictive model

The 5-year PFS probability model derived from multivariable Cox analysis had a C-index of 0.874 (95% CI, 0.851–0.898) in the training cohort and 0.769 (95% CI, 0.724–0.813) in the external validation cohort. Time-dependent ROC curves for the 5-year PFS probability model were plotted for both the training and validation cohorts, as shown in **Figure 5**. The area under the curve for the 5-year PFS probability was 0.897 (95% CI, 0.843–0.951) in the training cohort and 0.800 (95% CI, 0.687–0.914) in the external validation cohort.

**Figure 5:**
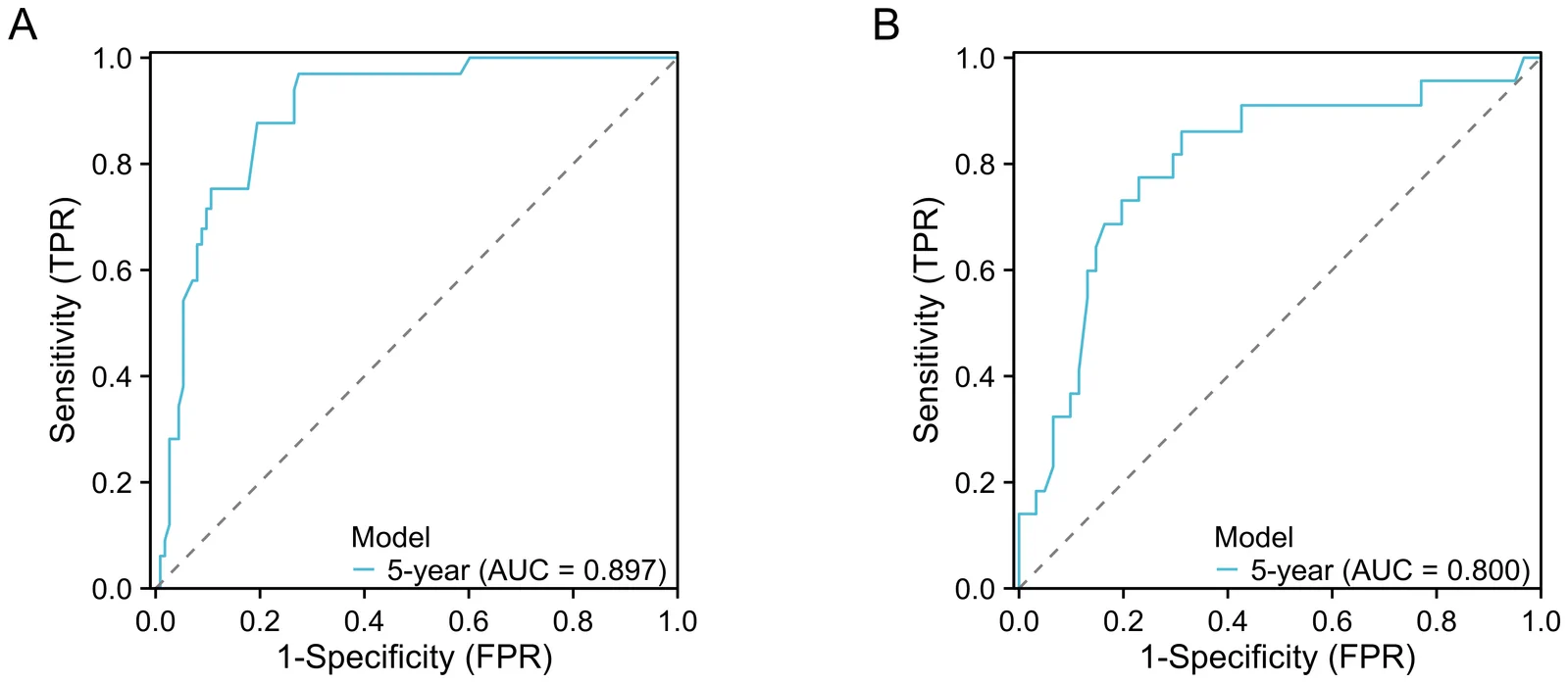
Time-dependent receiver operating characteristic (ROC) curves for the 5-year PFS probability nomogram in patients with DTC-LM. **(A)** Training cohort. **(B)** external validation cohort. PFS, progression-free survival; DTC, differentiated thyroid cancer; LM, lung metastases; ROC, receiver operating characteristic; AUC, area under the ROC curve.

Calibration of the prognostic model was evaluated by comparing predicted probabilities with observed event rates at consecutive time points, as depicted by the calibration curve. Calibration performance is reflected by the closeness of the curve to the ideal reference line. A deviation from the diagonal line indicates miscalibration, suggesting reduced predictive accuracy of the model. The calibration curve for the 5-year PFS probability model is shown in **Figure 6**, demonstrating close agreement between predicted probabilities and observed event incidence at various time points in both the training and validation cohorts. In addition, DCA confirmed the satisfactory clinical net benefit of the nomogram for predicting the 5-year PFS probability in DTC-LM patients (**Figure 7**).

**Figure 6:**
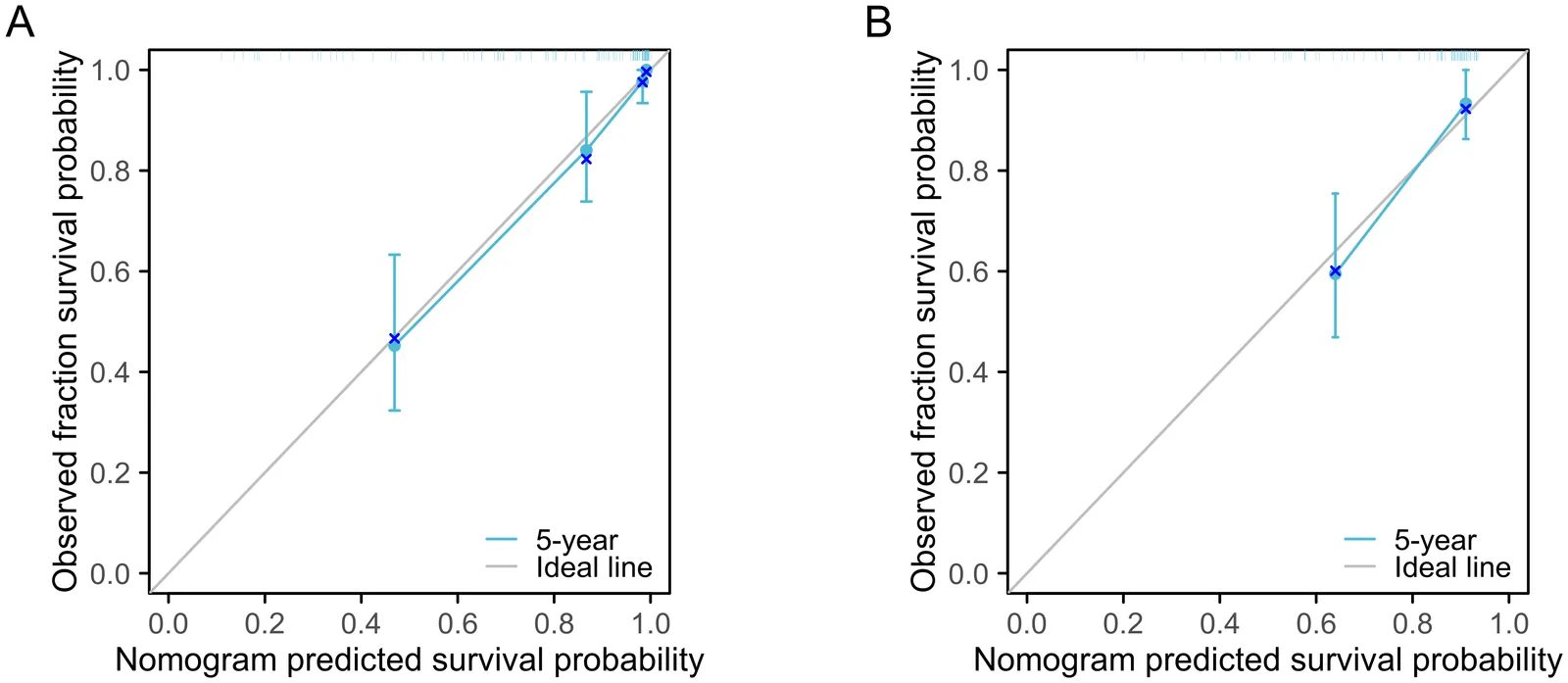
Calibration curves for the 5-year PFS probability nomogram in patients with DTC-LM. **(A)** Training cohort. **(B)** external validation cohort. The x-axis represents the nomogram-predicted survival probability, and the y-axis represents the observed fraction survival probability. The grey diagonal line indicates the ideal reference line. PFS, progression-free survival; DTC, differentiated thyroid cancer; LM, lung metastases.

**Figure 7:**
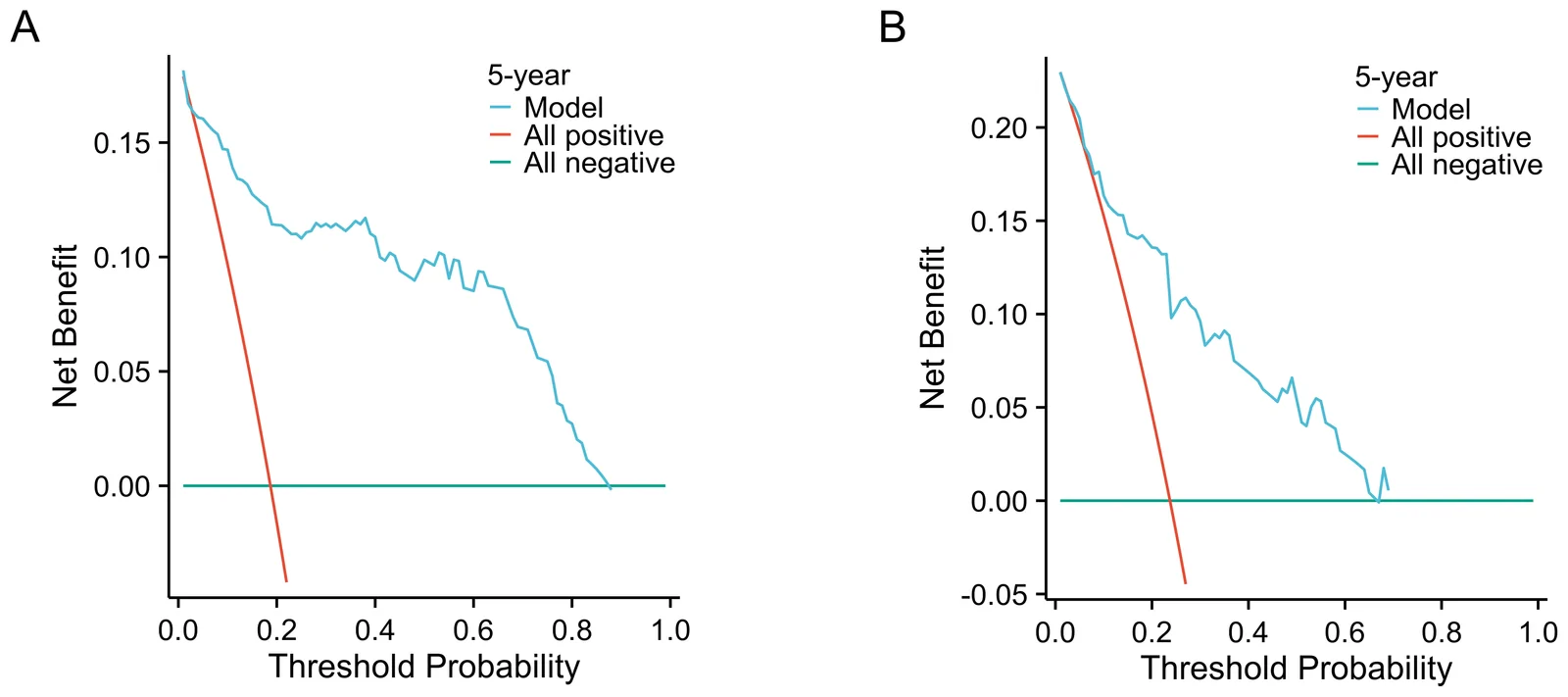
Decision curve analysis (DCA) of the 5-year PFS probability nomogram in patients with DTC-LM. **(A)** Training cohort. **(B)** external validation cohort. The y-axis represents net benefit, and the x-axis represents threshold probability. PFS, progression-free survival; DTC, differentiated thyroid cancer; LM, lung metastases; DCA, decision curve analysis.

## 4 Discussion

In this retrospective study, we developed and validated a nomogram for predicting the 5-year PFS probability in DTC-LM patients. Our model incorporated four independent risk factors identified through multivariate Cox regression: age upon diagnosis, tumor size, Ps-Tg, and initial RAI avidity status. The nomogram demonstrated excellent predictive performance, with a C-index of 0.874 in the training cohort and 0.769 in the external validation cohort, suggesting robust discriminative ability for identifying DTC-LM patients at high risk of the 5-year PFS.

Because follow-up duration varied across patients and PD was assessed longitudinally, we used Cox proportional hazards regression rather than a binary logistic model. This time-to-event approach accounts for censoring and differences in time to progression, and the nomogram estimates a fixed-time (5-year) PFS probability, which is clinically meaningful for treatment decision-making.

Age upon diagnosis emerged as one of the strongest independent predictors of PD in our study, consistent with findings from multiple previous investigations (11, 21, 22). The prognostic significance of age in DTC has been well established, with the AJCC/TNM staging system adopting 55 years as a key stratification threshold in its 8th edition (23). Our results align with those of Ji et al. (24), who demonstrated that the survival gap was greatest between patients aged <55 years and those aged ≥55 years in pulmonary metastatic DTC. Similarly, in a cohort of 89 DTC patients with pulmonary metastases, Sohn et al. (25) identified age ≥55 years as an independent predictor of poor prognosis. Relative to younger patients (<55 years), those aged ≥55 years had significantly inferior progression-free survival and cancer-specific survival. The consistent association between advancing age and worse outcomes across different studies underscores age as a critical factor in risk stratification for DTC-LM patients.

Serum Tg is a well‐established biomarker produced by thyroid tissue and DTC lesions, recognized for its convenience, reproducibility, and sensitivity in postoperative monitoring, particularly after thyroid remnant ablation (3). The clinical value of serum Tg measurements for initial risk stratification, treatment planning, and prognostic evaluation has been substantiated by multiple studies (26–28). In our study, an Ps-Tg level ≥528.5 ng/mL emerged as an independent predictor of PD for DTC-LM patients. The prognostic significance of Tg is closely linked to its reflection of overall tumor burden, indeed, each gram of neoplastic thyroid tissue can raise serum Tg by approximately 1 mg/L during thyroxine therapy, and by 2–10 mg/L following TSH stimulation (17). Previous studies have demonstrated that increasing Tg levels are associated with tumor progression, particularly in patients with RAIR disease or distant metastases (9).Our identified Ps-Tg threshold of 528.5 ng/mL thus offers a clinically actionable benchmark for detecting DTC-LM patients at elevated risk of progression, enabling timely consideration of alternative therapeutic strategies such as MKIs.

Initial RAI avidity status emerged as the independent predictor of PD in our model, with partial or no RAI avidity conferring a markedly elevated risk (HR: 8.462). This finding is consistent with a growing body of evidence underscoring the critical role of RAI avidity in determining clinical outcomes in DTC-LM patients. Similarly, Qiu et al. (29) reported that loss of ¹³¹I avidity was the strongest independent predictor of poor prognosis and survival in PTC patients with lung metastases during long-term follow-up. A study of patients with three or more distant organ system metastases further confirmed that RAI avidity and RAIR-DTC were the main independent prognostic factors influencing clinical outcomes (30).

The biological basis for this association lies in tumor dedifferentiation, a process characterized by downregulation of thyroid-specific molecules, particularly the sodium-iodide symporter (NIS), which is critical for RAI uptake (9, 17, 31). During the natural disease course or following repeated RAI therapy, approximately one-third of DTC-LM patients exhibit functional regression with loss of RAI avidity, ultimately progressing to RAIR disease. Once RAIR develops, these patients no longer benefit from RAI therapy and face a dramatically poorer prognosis, with a 10-year survival rate of approximately 10% (25). Importantly, the heterogeneity of RAI avidity patterns further stratifies prognosis: patients whose lesions never concentrate RAI, those who gradually lose avidity, and those with partial uptake all exhibit distinct clinical trajectories. Our findings underscore that initial RAI avidity status is not merely a binary classification but a dynamic indicator of tumor biology that should guide clinical decision-making. For patients with partial or no RAI avidity, early consideration of alternative therapeutic strategies such as MKIs is warranted, as emphasized in recent guidelines recommending lenvatinib or sorafenib as first-line therapy for progressive RAIR-DTC (3, 9).

The nomogram approach offers several advantages over conventional staging systems. Unlike the AJCC/TNM system, which primarily predicts disease-specific mortality, our nomogram provides an individualized, quantitative estimate of the 5-year PFS probability based on readily accessible clinical and serological parameters (24). This is particularly valuable in the DTC-LM population, where timely identification of patients at risk of progression can guide clinical decision-making regarding the initiation of alternative therapies and the avoidance of ineffective repeated RAI therapy. The user-friendly nature of our nomogram, requiring only age upon diagnosis, tumor size, Ps-Tg, and initial RAI avidity status, enhances its applicability in routine clinical practice, especially in resource-limited settings where expensive molecular imaging modalities such as ^18^F-FDG PET/CT may not be universally available.

Several limitations should be acknowledged. First, this was a retrospective study with inherent selection bias. Second, although the model was validated in an independent external cohort from another institution, the external validation cohort was still from a single center, and further prospective multicenter validation is warranted. Third, the Ps-Tg cutoff of 528.5 ng/mL was derived from ROC analysis in the training cohort used for model development, which may introduce optimism. Fourth, genetic profiling data such as TERT promoter mutations were not included and may provide additional predictive value. Fifth, follow-up duration remained heterogeneous, and the 5-year prediction should be interpreted with caution in patients with shorter observation periods.

Despite these limitations, our nomogram represents a valuable tool for risk stratification in DTC-LM patients. By providing an individualized, clinically applicable prediction of the 5-year PFS probability, it may facilitate early identification of high-risk patients, enabling timely therapeutic interventions and ultimately improving clinical outcomes. Future prospective studies incorporating molecular biomarkers and external validation will be essential to further refine and validate this predictive model.

## 5 Conclusion

In conclusion, older age upon diagnosis, larger tumor size, partial or no RAI avidity, and elevated Ps-Tg levels were identified as independent predictors of PD in DTC-LM patients. A 5-year PFS probability nomogram integrating these four risk factors showed good discrimination and favorable net clinical benefit. However, given the retrospective design, modest sample size, and single-center external validation, the model should be regarded as a preliminary risk-stratification tool, and further prospective multicenter validation is warranted.

## Data Availability

The original contributions presented in the study are included in the article/supplementary material. Further inquiries can be directed to the corresponding author.
